# Investigating SOFA, delta-SOFA and MPM-III for mortality prediction among critically ill patients at a private tertiary hospital ICU in Kenya: A retrospective cohort study

**DOI:** 10.1371/journal.pone.0235809

**Published:** 2020-07-16

**Authors:** Lillian N. Lukoko, Peter S. Kussin, Rodney D. Adam, James Orwa, Wangari Waweru-Siika

**Affiliations:** 1 Department of Anesthesia, Aga Khan University Hospital, Nairobi, Kenya; 2 Division of Pulmonary and Critical Care Medicine, Duke University, Durham, North Carolina, United States of America; 3 Departments of Pathology and Medicine, Aga Khan University Hospital, Nairobi, Kenya; 4 Department of Population Health, Aga Khan University Hospital, Nairobi, Kenya; National Yang-Ming University, TAIWAN

## Abstract

**Background:**

Outcomes in well-resourced, intensive care units (ICUs) in Kenya are thought to be comparable to those in high-income countries (HICs) but risk-adjusted mortality data is unavailable. We undertook an evaluation of the Aga Khan University Hospital, Nairobi ICU to analyze patient clinical-demographic characteristics, compare the performance of Sequential Organ Failure Assessment (SOFA), delta-SOFA at 48 hours and Mortality Prediction Model-III (MPM-III) mortality prediction systems, and identify factors associated with increased risk of mortality.

**Methods:**

A retrospective cohort study was conducted of adult patients admitted to the ICU between January 2015 and September 2017. SOFA and MPM-III scores were determined at admission and SOFA repeated at 48 hours.

**Results:**

Approximately 33% of patients did not meet ICU admission criteria. Mortality among the population of critically ill patients in the ICU was 31.7%, most of whom were male (61.4%) with a median age of 53.4 years. High adjusted odds of mortality were found among critically ill patients with leukemia (aOR 6.32, p<0.01), tuberculosis (aOR 3.96, p<0.01), post-cardiac arrest (aOR 3.57, p<0.01), admissions from the step-down unit (aOR 3.13, p<0.001), acute kidney injury (aOR 2.97, p<0.001) and metastatic cancer (aOR 2.45, p = 0.04). The area under the receiver-operating characteristic (ROC) curve of admission SOFA was 0.77 (95% CI, 0.73–0.81), MPM-III 0.74 (95% CI, 0.69–0.79), delta-SOFA 0.69 (95% CI, 0.63–0.75) and 48-hour SOFA 0.83 (95% CI, 0.79–0.87). The difference between SOFA at 48 hours and admission SOFA, MPM-III and delta-SOFA was statistically significant (chi^2^ = 17.1, 24.2 and 26.5 respectively; p<0.001). Admission SOFA, MPM-III and 48-hour SOFA were well calibrated (p >0.05) while delta-SOFA was borderline (p = 0.05).

**Conclusion:**

Mortality among the critically ill was higher than expected in this well-resourced ICU. 48-hour SOFA performed better than admission SOFA, MPM-III and delta-SOFA in our cohort. While a large proportion of patients did not meet admission criteria but were boarded in the ICU, critically ill patients stepped-up from the step-down unit were unlikely to survive. Patients admitted following a cardiac arrest, and those with advanced disease such as leukemia, stage-4 HIV and metastatic cancer, had particularly poor outcomes. Policies for fair allocation of beds, protocol-driven admission criteria and appropriate case selection could contribute to lowering the risk of mortality among the critically ill to a level on par with HICs.

## Introduction

Intensive care mortality rates in low and middle-income countries (LMICs) are significantly higher than those in high-income countries (HICs), ranging between 35.1% and 53.6% [1–5]. The higher mortality rates in Africa in particular have been attributed to a high critical illness burden. Worldwide consensus is that this burden and subsequently high mortality is due to the synergism that exists between higher resource-limitation, poor nutrition, trauma, the burden of neglected chronic illnesses, poor access to care, late presentation to critical care and delayed evaluation [1, 6–9].

Kenya is a lower middle-income country [10] with a population of 47.6 million and expenditures for healthcare at 169 US dollars per capita, or about 6% of GDP [11, 12]. The bulk of healthcare in Kenya is government-subsidized, and institutions are classified from Level 1 (community hospitals) to Level 6 (national referral hospitals), based on resource level [13]. Approximately 25 million Kenyans are covered under the National Health Insurance Fund scheme, a government-funded health insurer subsidizing care in Kenya [14]. In contrast, private and faith-based institutions meet a much smaller proportion of the healthcare needs of Kenyans, predominantly offering services to fee-paying patients. Thirty-two private health insurers provide coverage for only 17% of all Kenyans, 27% of the urban population and up to 42% of Kenya’s wealthy [8].

As in many parts of Sub-Saharan Africa, there is substantial heterogeneity in resources consistently available in the ICU. The national referral hospital ICUs are predominantly managed by anesthesiologists, but staffing may vary across smaller county and private hospital ICUs [15]. Core ICU services such as mechanical ventilation, inotropic support, invasive neuro-monitoring and bedside renal replacement therapy, and supporting infrastructure such as pathology and laboratory services, are not consistently available across the country [15, 16].

The Aga Khan University Hospital Nairobi (AKUHN) is a private 280-bed Level 6 facility situated in the capital city of Nairobi, accredited by the Joint Commission International since 2013 for its quality of care [17]. The hospital offers a wide range of specialized outpatient and inpatient services that span the spectrum of medical and surgical specialties, with state-of-the-art pathology, laboratory and radiological services readily available. AKUHN serves patients from Kenya as well as other East and Central African countries, with medical care for most financed through private medical insurance or cash payment. The hospital also has a charity program which supports eligible patients without financial means to pay for their care.

No study to date has assessed ICU outcomes in well-resourced private institutions in Kenya that provide care at standards equivalent to the best academic tertiary care teaching facilities in HICs. Benchmarking efforts are hampered by insufficient evidence to support the use of existing severity of illness scoring systems to guide the generation of risk-adjusted mortality data. Severity of illness scoring may be performed once at admission, daily or at given intervals during the course of care [18–20]. Differences in severity of illness scores between two clinical timepoints have been used to predict mortality, with conflicting results [18]. The aim of this study was to analyze patient clinical-demographic characteristics among patients admitted to the AKUHN ICU, compare the performance of Sequential Organ Failure Assessment (SOFA), delta-SOFA at 48 hours (i.e. the difference between admission SOFA and SOFA at 48 hours) and Mortality Prediction Model-III (MPM-III) mortality prediction systems, and identify factors associated with increased risk of mortality.

## Methods

This was a retrospective cohort study, conducted at the AKUHN ICU, an 11-bed mixed general, open unit with approximately 450 admissions per year. SOFA scoring at admission and daily thereafter has been performed here since 2016. The unit is staffed by a multidisciplinary team of physicians, including full-time critical care physicians, 85% of whom had formal training in intensive care at the time of this study. Being an open ICU, care is coordinated by the admitting physician or surgeon, with intensivists playing a predominantly advisory role. The nurse-to-patient ratio in the AKUHN ICU ranges between 1:1 and 1:2 based on staff availability, and 30–50% of ICU nurses have a Higher National Diploma in intensive care nursing.

The unit provides both invasive and non-invasive mechanical ventilation, invasive hemodynamic monitoring, inotropic support, invasive neuro-monitoring and bedside renal replacement therapy including continuous renal replacement therapy (CRRT). Evidence-based protocols and pathways guide the full spectrum of intensive care, adherence to which is monitored by hospital leadership. Paper records are maintained at this hospital, in addition to an electronic medical record system that is mainly used for laboratory and radiology data.

Housed within AKUHN and staffed by the same team of physicians is a mixed, general, 16-bed step-down unit (the high dependency unit) for lower acuity patients, averaging 1,500 admissions annually. Nurse-to-patient ratios here are 1:2, stretching to 1:3 occasionally depending on staff availability. Admission to the AKUHN ICU is limited to patients in need of multiorgan support and/or invasive ventilation, whereas the step-down unit admits those with single organ failure only. Despite having clear ICU and step-down unit admission criteria however, patients are often admitted to a unit with a higher or lower level of care than required (ICU instead of a step-down unit and vice versa), depending on bed availability, the frequency of which is audited. In addition, the institution does not have a policy for withholding ICU care from terminally ill or palliative care patients, except where an advanced directive is in place, or the legal next of kin decline such care.

Our primary objective was to analyze the difference in the area under the receiver-operating characteristic (ROC) curves of the scoring systems under investigation. Secondary objectives were to evaluate ICU mortality and to investigate the association between clinical and demographic factors and mortality among the critically ill. Patients who died or were discharged before 48 hours were excluded from delta-SOFA calculations but included in MPM-III and SOFA calculations. A minimum sample size of 677 critically ill patients was required to give the study 80% power to detect a difference of at least 10% between the area under the ROC curves of SOFA, delta-SOFA and MPM-III [21–23], adjusting for an estimated 40% of patients who would die or be discharged from the ICU within 48 hours (based on the 2016 AKUHN ICU admission register), with a p-value of < 0.05.

The ICU admission register was used to identify patients admitted to the ICU, and their files retrieved by the medical records team. Consecutive sampling was performed until the desired sample size was achieved. A comprehensive review of patient files was conducted, and clinical and demographic variables entered into a spreadsheet using Microsoft Office ® Excel 2010 (Redmond, WA, USA). Where files were not available for review, a minimum dataset consisting of age, sex, admission and discharge data was extracted from the ICU admission register. Patients admitted with burns, those for cardiac surgery and readmissions during the same hospitalization were excluded, as is standard practice for the MPM-III scoring system. Patients who were boarding in the ICU due to unavailability of beds in the general wards and overflow from the step-down unit were excluded from analyses of ICU outcomes as they did not meet the pre-specified ICU admission criteria.

Demographic and outcome variables were analyzed as percentages for categorical variables and medians with interquartile ranges for continuous variables. Non-parametric data for other outcomes were analyzed as medians (interquartile ranges) or means (standard deviation) for parametric data. Chi square or Fisher’s exact tests were used to evaluate risk factors independently associated with mortality and multivariate logistic regression analysis used to control for confounders. Missing data for SOFA and MPM-III were assumed to be normal and absent respectively, as is standard practice with these scoring systems [24]. SOFA scores were calculated by totaling the values obtained for each variable. Delta-SOFA at 48-hours was obtained by deducting the SOFA score at 48 hours from admission SOFA. MPM-III scores were calculated using an online scoring calculator. Data was entered into an MS Excel® spreadsheet.

The area under the ROC curve was used to evaluate the discrimination of the models. An ROC curve with an area of 0.7–0.8 was considered fair, 0.8–0.9 good and >0.9 excellent [21]. The area under the ROC curves were compared using non-parametric Wilcoxon statistics. A Hosmer-Lemeshow goodness-of-fit test was used to assess the calibration of the models with a p-value of >0.05 considered statistically significant. For all other statistical tests, a p-value of < 0.05 was considered statistically significant. All analyses were performed using Stata/SE 14.2 (StataCorp LP, College Station, TX). Ethical clearance was obtained from the AKU Institutional Ethics Review Committee (IERC) prior to commencement (IERC Approval Reference 2017/REC-56).

## Results

**Fig 1:**
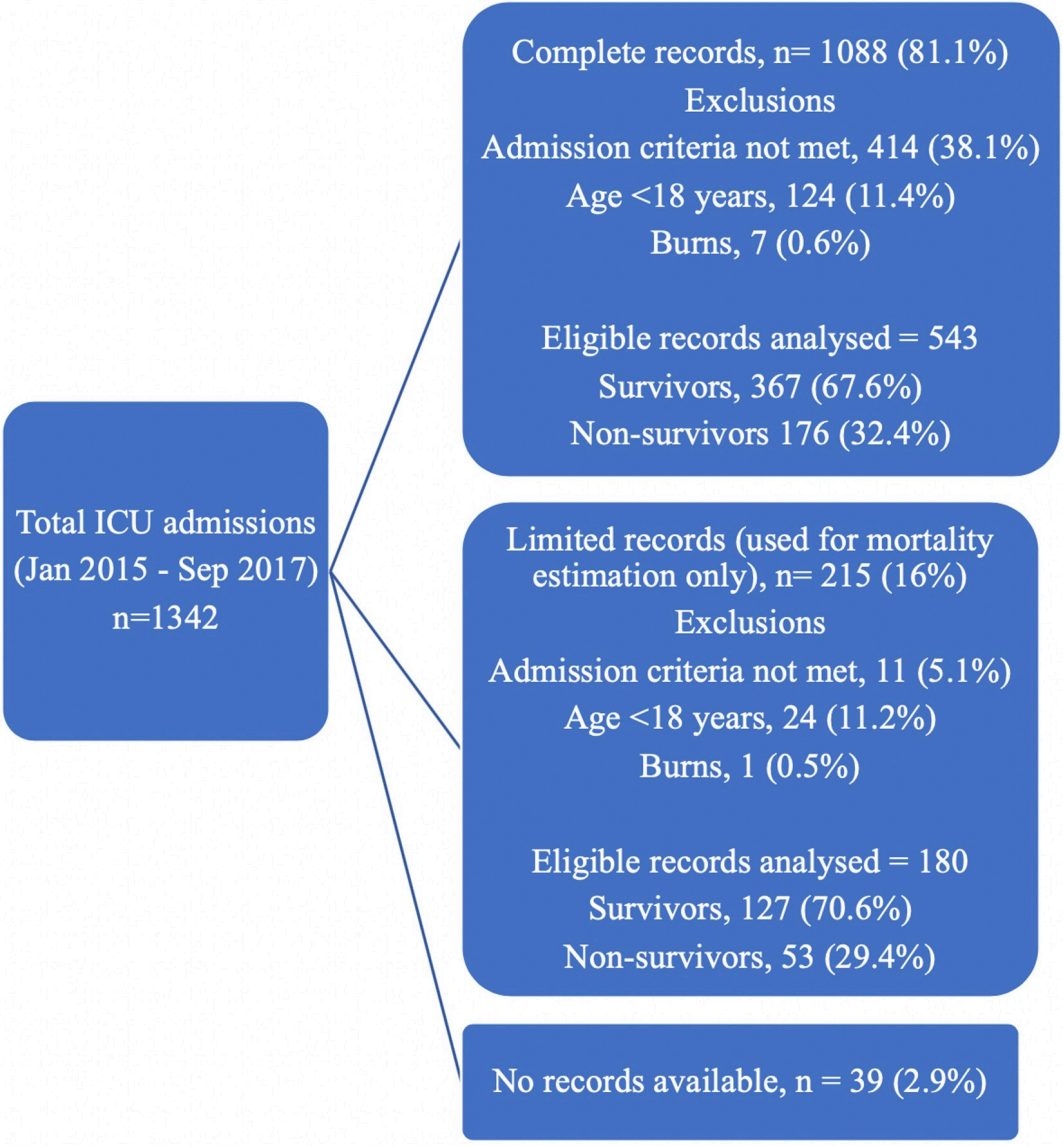
CONSORT diagram.

There were 1,342 admissions to the AKUHN ICU between January 2015 and September 2017, 1,088 (81.1%) of whom had complete records available for review (Fig 1). A further 215 had limited records only i.e. had missing sections of files, 179 of whom were eligible for inclusion. Demographic and survival data for this group was extracted from the ICU admission register. For 39 patients, records could not be retrieved due to errors in names and/or identification numbers, rendering the patient unidentifiable. Overall, 425 (32.6%) of 1,303 patients with limited or complete records available for review did not meet ICU admission criteria.

Data from 723 critically ill patients of the 1,342 admissions to the ICU during the study period were analyzed (53.9% of all admissions). Most of these were male (61.4%), with a median age of 53 years (Table 1). Among the 543 critically ill patients with complete records available for review, medical patients formed the bulk of admissions (61.3%). The majority of surgical patients were emergency admissions (78%), with neurosurgical cases comprising the single largest category amongst all surgical admissions at 61 (29%). The majority of critically ill patients admitted to the ICU were not screened for HIV, although 44% of those who were screened were found to be HIV positive.

**Table 1:** Clinical-demographic characteristics of critically ill patients at admission.

Variable	Frequency (%)	
**Gender, n = 722**^a^		
Male	444 (61.4)	
Female	278 (38.5)	
Median (IQR) age in years	53 (39–69)	
**Origin prior to hospital admission, n = 543**		
Home	353 (65.0)	
Another facility	165 (30.4)	
Accident site	25 (4.6)	
**Origin prior to ICU admission, n = 543**		
Step-down unit	115 (21.2)	
Theatre	131 (24.1)	
Accident and emergency	217 (40.0)	
Ward	52 (9.6)	
Coronary care unit	17 (3.1)	
Other^b^	11 (2.0)	
**Type of admission, n = 543**		
Medical	333 (61.3)	
Surgical	210 (38.7)	
**HIV status, n = 543**		
Positive	41 (7.6)	
Negative	52 (9.6)	
Unknown	450 (82.9)	
**Severity of illness scores at admission**		
Scoring system	Mean (SD^c^)	Median (IQR^d^)
Admission SOFA (SOFA_0_)	6.9 (4.2)	6 (4–9)
MPM-III	28.5 (26.6)	19.4 (5.5–47.8)

^a^Data missing for one patient;

^b^Other admissions were from the coronary care unit (n = 17), cardiac catheterization lab (n = 7), endoscopy unit (n = 2), chemotherapy unit (n = 1) and renal unit (n = 1); ^c^SD, standard deviation; ^d^IQR, interquartile range

Most critically ill patients had more than one indication for ICU admission, with 51(9.4%) admitted following a cardiac arrest (Table 2). The most common indications for ICU admission were respiratory failure in 177 (32.6%), acute kidney injury in 99 (18.2%) and sepsis or septic shock in 95 (17.5%) of 543 critically ill patients.

**Table 2:** Indication for ICU admission among critically ill patients, n = 543.

Indication	Frequency (%)
**Cardiovascular system**	
Post-cardiac arrest	51 (9.4)
**Respiratory system**	
Pneumonia	88 (16.2)
Respiratory failure	177 (32.6)
Pulmonary edema	32 (5.9)
ARDS^a^	29 (5.3)
**Central nervous system**	
Intracranial hematoma	39 (7.2)
Subarachnoid hemorrhage	34 (6.3)
Intracranial tumors	32 (5.9)
Traumatic brain injury (TBI)	20 (3.7)
**Gastrointestinal disease**	
Gastrointestinal bleed	12 (2.2)
Acute pancreatitis	14 (2.6)
Hepatic encephalopathy	9 (1.7)
**Obstetrics**	
Post-partum hemorrhage (PPH)	4 (0.7)
Pre-eclampsia/Eclampsia	2 (0.4)
**Renal disease**	
Acute kidney injury (AKI)	99 (18.2)
**Intoxication**	
Poisoning	7 (1.3)
**Surgical conditions**	
Post-explorative laparotomy	30 (5.5)
Post-craniotomy	61 (11.2)
Other surgical conditions	119 (21.9)
**Others**	
Sepsis	39 (7.2)
Septic shock	56 (10.3)

^a^Acute Respiratory Distress Syndrome

More than one co-morbidity per critically ill patient were recorded where present (Table 3). The most frequent co-morbid conditions were hypertension (27.5%) and diabetes mellitus (11.4%). The difference between survivors and non-survivors among critically ill patients with chronic obstructive pulmonary disease (COPD), ischemic heart disease, heart failure, hypertension, diabetes and chronic kidney disease was not statistically significant.

**Table 3:** Comorbid conditions among critically ill patients admitted to the ICU, n = 543.

Comorbid condition	Prevalence Number (%)	Survivors, Number (%)	Non-survivors, Number (%)	p-value
Heart failure	21/547 (3.8)	13 (61.9)	8 (38.1)	0.66
Coronary artery disease	54/554 (9.8)	39 (72.2)	15 (27.8)	0.45
Hypertension	150/545 (27.5)	97 (64.7)	53 (35.3)	0.35
Asthma	23/544 (4.2)	16 (69.6)	7 (30.4)	0.31
COPD^a^	26/545 (4.8)	18 (69.2)	8 (30.8)	0.87
Chronic kidney disease	37/544 (6.8)	26 (70.3)	11 (29.7)	0.71
Diabetes mellitus	62/546 (11.4)	40 (64.5)	22 (35.5)	0.58
Leukemia	10/543 (1.8)	3 (30.0)	7 (70.0)	0.01
Metastatic cancer	25/544 (4.6)	12 (48.0)	13 (52.0)	0.03
HIV^b^	41/543 (7.6)	18 (43.9)	23 (56.1)	<0.01
Tuberculosis	22 (4.1)	8 (36.4)	14 (63.6)	0.001

^a^COPD, chronic obstructive pulmonary disease;

^b^HIV, Human Immunodeficiency Virus

Median predicted mortality among 723 critically ill patients with limited or complete records was 10–15% based on admission SOFA, and 19.4% based on MPM-III (IQR 5.5–47.8%). Observed mortality in this population was 229 (31.7%), with a median ICU length-of-stay (LOS) of 4 days (SD 11.9, IQR 1–9). Among the 543 critically ill patients with complete records, mortality was 32.4% (n = 176) with a median LOS of 4 days (SD 12.8, IQR 2–10). There was no difference in ICU LOS between survivors (4 days, SD 14.6, IQR 2–11) and non-survivors (4 days, SD 7.1, IQR 1–8). Overall, 190 (26.3%) of 723 critically ill patients were in the ICU for less than 48 hours, with 76 (40%) dead and 114 (60%) discharged to a lower level of care within that time.

A greater proportion of non-survivors than survivors was found among critically ill patients with leukemia, metastatic cancer, HIV and tuberculosis (Table 3). Mortality in HIV-infected critically ill patients was 56.1%, with 17 (41.5%) of those infected admitted with World Health Organization (WHO) stage-4 HIV infection. Mortality in this group was 64.7%.

Mechanical ventilation was instituted in 322 (66%) of 543 critically ill patients, 38% of whom survived, while vasopressors were administered in 110 (20%), 89% of whom survived. The highest mortality at 48-hours and overall was found in critically ill patients admitted to the ICU from the step-down unit, who also had higher SOFA scores at admission than the unit average (chi^2^ 41.2, p = 0.002) (Table 4).

**Table 4:** Mortality by origin, length of stay prior to ICU admission and severity of illness among critically ill patients admitted to the ICU.

Origin prior to ICU^a^ admission	Hospital LOS^b^ prior to ICU admission Median (IQR^c^)	Admission SOFA Median (IQR)	Delta-SOFA at 48 hours Median (IQR)	48-hr mortality by origin prior to ICU admission (no, %)	Overall mortality by origin prior to ICU admission (%)
A&E^d^, n = 217	NA^e^	6 (3–9)	0 (-2-+2)	27/55 (49.1%)	70/217 (32.3%)
Step-down unit, n = 115	2 (1–5)	9 (6–13)	0 (-2-+2)	14/18 (77.8%)	62/115 (53.9%)
^f^Other, n = 28	2.5 (0–8.5)	8 (6–11)	0 (-1-+3)	3/7 (42.9%)	11/28 (39.3%)
Theatre, n = 131	1 (0–2)	6 (3–7)	-1 (-3-0)	5/35 (14.3%)	17/131 (13%)
Ward, n = 52	3 (1–6.5)	5.5 (4–8)	0 (-1-+2)	6/13 (46.2%)	16/52 (30.8%)
Entire cohort, n = 543	1 (0–2)	6 (4–9)	0 (-2-+2)	76/190 (40.0%)	176/543 (32.4%)

^a^ICU, Intensive Care Unit;

^b^LOS, length-of-stay;

^c^IQR, interquartile range;

^d^A&E, accident and emergency;

^e^NA, not applicable;

^f^Other admissions: coronary care unit (n = 17), cardiac catheterization lab (n = 7), endoscopy unit (n = 2), chemotherapy unit (n = 1) and renal unit (n = 1)

SOFA points ≥2 for a given organ system were associated with increased odds of mortality among the critically ill: hematological system odds ratio, OR 4.79 (95% CI 1.90–12.04, p = 0.001), cardiovascular system, OR 3.58 (2.14–5.98, p = <0.001), central nervous system, OR 2.30 (1.49–3.55, p = <0.001) and respiratory system, OR 1.68 (1.11–2.53, p = 0.014). Median delta-SOFA was 0 (IQR -2-+2). A baseline SOFA score of 16 or more at admission among critically ill patients admitted to the ICU was associated with 100% mortality while 74 (47.7%) of 155 patients with a rising SOFA score at 48 hours died, OR 4.5 (95% CI 2.9–7.2, p<0.001).

Critically ill patients with leukemia, tuberculosis, post-cardiac arrest, acute kidney injury (AKI), sepsis, metastatic cancer and stage-4 HIV had the highest adjusted odds for mortality (Table 5). Mean admission SOFA for patients with leukemia was 9 (median 8, SD 5.7, IQR 4–12), tuberculosis 8.3 (median 8.5, SD 3.8, IQR 6–12), post-cardiac arrest 11 (median 11, SD 3.7, IQR 9–13), AKI 10.6 (median 10, SD 4.2, IQR 7–13), sepsis 9.5 (median 10, SD 4.5, IQR 6–13), metastatic cancer 8.8 (median 8, SD 4.4, IQR 6–11.5), HIV infection 8.5 (median 7,SD 4.4, IQR 5–11) and stage-4 HIV disease 10.4 (median 11.5, SD 4.1, IQR 6.5–13). The adjusted OR for mortality among critically ill HIV-infected patients (n = 41), and those with WHO stage-4 disease (n = 16) was not statistically significant.

**Table 5:** Factors associated with increased odds of mortality among critically ill patients.

Variables	Unadjusted odds ratios			Adjusted odds ratios		
	Odds Ratio	p-value	95% CI^a^	Odds Ratio	p-value	95% CI
Age (yrs), n = 722	1.01	0.01	1.00–1.02	1.35	0.01	1.00–1.02
Male, n = 542	1.42	0.07	0.98–2.07	1.01	0.06	0.98–2.10
Origin prior to ICU^b^ admission, n = 543						
Step-down unit	3.22	<0.001	2.10–4.92	3.13	<0.001	2.04–4.82
Other^c^	1.42	0.38	0.65–3.07	1.27	0.56	0.58–2.79
Length-of-stay (LOS) (days)						
Hospital LOS prior to ICU admission	1.04	0.01	1.00–1.08	1.04	0.01	1.01–1.07
Diagnosis at ICU admission, n = 543						
Leukemia	5.03	0.02	1.28–19.67	6.32	0.01	1.59–25.16
Tuberculosis	3.89	<0.01	1.60–9.45	3.96	<0.01	1.62–9.67
Post-cardiac arrest	3.54	<0.001	1.97–6.37	3.57	<0.001	1.94–6.57
Acute kidney injury	3.21	<0.001	2.06–5.0	2.97	<0.001	1.88–4.71
Sepsis	3.16	<0.001	2.02–4.95	2.85	<0.001	1.80–4.52
HIV^d^	2.63	0.03	1.13–6.13	2.48	0.05	1.01–6.11
Stage-4 HIV	3.49	0.02	1.23–9.94	2.63	0.10	0.83–8.34
Metastatic cancer	2.37	0.04	1.06–5.30	2.45	0.04	1.04–5.76
Respiratory failure	2.16	<0.001	1.48–3.15	2.22	<0.001	1.51–3.24
Vital signs on admission to ICU, n = 543						
Respiratory rate (breaths/min)	1.06	<0.001	1.04–1.09	1.07	<0.001	1.04–1.10
Heart rate (beats/minute)	1.02	<0.001	1.02–1.03	1.02	<0.001	1.02–1.03

^a^CI, confidence interval;

^b^ICU, Intensive Care Unit;

^c^Other admissions: coronary care unit (n = 17), cardiac catheterization lab (n = 7), endoscopy unit (n = 2), chemotherapy unit (n = 1) and renal unit (n = 1);

^d^HIV, Human Immunodeficiency Virus

The area under the ROC curve of admission SOFA was 0.77 (95% CI, 0.73–0.81), MPM-III 0.74 (95% CI, 0.69–0.79), delta-SOFA 0.69 (95% CI, 0.63–0.75) and 48-hour SOFA 0.83 (95% CI, 0.79–0.87) among critically ill patients (Table 6). The difference between SOFA at 48 hours and admission SOFA, MPM-III_0_ and delta-SOFA in this population was statistically significant (chi^2^ = 17.1, 24.2 and 26.5 respectively, p<0.001). Admission SOFA, MPM-III and 48-hour SOFA were well calibrated (p >0.05) while delta-SOFA was borderline (p = 0.05).

**Table 6:** Discrimination and calibration of SOFA, delta-SOFA and MPM-III among critically ill patients admitted to the ICU.

Risk prediction model	SOFA_0_^a^ (n = 543)	MPM-III (n = 543)	SOFA_48_^b^ (n = 406)	delta-SOFA (n = 406)
Area under the ROC^c^ curve	0.77	0.74	0.83	0.69
Hosmer-Lemeshow chi-square statistic	9.37	8.91	4.73	12.57
Hosmer-Lemeshow p-value	0.23	0.35	0.79	0.05

^a^SOFA_0_, Admission SOFA score;

^b^SOFA_48_, SOFA score at 48-hours;

^c^ROC, Receiver-operating characteristic

## Discussion

The objectives of this retrospective study were twofold. Our primary objective was to examine the performance and utility in our setting of three commonly used mortality prediction models. Our results show that while admission SOFA and MPM-III had fair discrimination and were well calibrated among critically ill patients admitted to the AKUHN ICU, delta-SOFA performed poorly. SOFA at 48-hours had the best overall performance in this cohort. While the area under the ROC curve for MPM-III in our study was similar to that from a study from Rwanda [4], it was better calibrated in our population of critically ill patients. Calibration has been shown to be affected by hospital management policies, patient case mix and critical care interventions, and may account for the difference between our two populations [25]. The poor performance of delta-SOFA at 48-hours for mortality prediction despite 48-hour SOFA’s good discrimination in our cohort may be attributed to the predominance of unchanged high SOFA scores at 48 hours, [18, 19, 26].

Our second objective was to identify factors associated with higher risk of mortality among critically ill patients in a well-resourced private ICU in a lower middle-income country. We found that mortality was surprisingly high for such a well-resourced ICU. The crude mortality rate of 31.7% among the population of critically ill patients admitted to the AKUHN ICU was substantially greater than the reported range of 8–19% in HICs [27–30]. While this was significantly lower than in the 53.6% mortality in a public ICU in Kenya [5], it was higher than the 26.6% mortality in of our faith-based institutions [30]. This finding was somewhat unexpected given that the AKUHN ICU is significantly better-resourced than most facilities in Kenya, comparable in staffing and infrastructure to facilities in the United States and Europe. With such discrepant ICU outcomes from studies conducted in Kenya, closer inspection of these heterogeneous outcomes is warranted.

The public ICU cohort was younger (median age 29 years, IQR 15–47) compared to our critically ill (median age 53 years, IQR 39–69) [5]. The public ICU and our cohort were predominantly male (61% and 61.4% respectively). In the public ICU mortality was higher than both our institution and faith-based facilities (53.6% versus 31.7% versus 25.5% respectively). In the public ICU the majority (61.6%) were transferred-in from other healthcare facilities, which was associated with higher mortality compared to those who came from home (56.7% vs 47.9%); the majority of our critically ill (65%) came directly from home. In general, the faith-based cohort was comprised of low-acuity patients mainly, admitted predominantly for monitoring, with only 22% requiring mechanical ventilation—a basic indicator of critical care severity. This is in sharp contrast to the high utilization of mechanical ventilation in our cohort and in the public ICU group.

Our results also highlight the impact of HIV in critical care in Kenya and on the outcomes of critical care in our region reported here and elsewhere. An analysis of critical care outcomes in LMICs would be incomplete without a discussion of outcomes in HIV. In 2018, 1.6 million people were living with HIV, with a prevalence in adults of 4.9%, and 28,200 people had died from an AIDS-related illness [31]. While a greater proportion of critically ill patients in the AKUHN ICU were HIV-infected compared to those in the public ICU (44% vs 13.1%), our overall mortality in this population of patients was lower. Mortality among HIV-infected critically ill patients in our cohort was high (56.1%), with 64.7% mortality in those with WHO stage-4 HIV infection. Although this was not statistically significant, the numbers were small as only 18% patients were screened for HIV, a low rate given that Kenya has an opt-out policy for HIV screening. A public facility ICU in Kenya however recorded HIV rates of only 13.1% [5] even when a larger proportion of patients (46%) were screened for HIV [5]. Undetected HIV infection is therefore unlikely to have significantly affected our results, given the ready availability of infectious disease specialists at our institution competent to detect clinical features suggestive of HIV infection, coupled with our low threshold for testing when the index of suspicion is high.

Limiting ICU care for those unfortunate patients who will not benefit from critical care at the end of life is a worldwide problem but one which is greatly accentuated in both the public and private sectors in Kenya and possibly worsened by the relatively rapid expansion of critical care in this country [16]. A fair allocation system seeking to limit ICU care for those with extremely poor prognosis would likely lead to a reduction in observed mortality at our institution and across Kenya. We have identified co-morbid conditions associated with higher mortality among the critically ill which are consistent across ICU settings in LMICs. These factors include admission following a cardiac arrest, acute leukemia, metastatic cancer, advanced HIV, and tuberculosis. All of these co-morbidities had higher SOFA scores than the unit average at admission. The ability to discuss limitations of critical care and life support are severely hampered by the absence of a framework, much less a consensus amongst caregivers and the public in Kenya, to implement policies to limit or withdraw intensive care from those who are at the end of life [16]. In a religious and cultural environment where the focus is on faith and hope, we do not see an easy path to public consensus regarding limiting or withdrawing life support in our ICUs.

Looking beyond patients admitted to critical care with marginal expected benefit, we make note of a subset of patients which indirectly contribute to poorer outcomes [32, 33]. In our “open model” system, patients are often admitted to the ICU or the step-down unit who could be cared for in a lower level setting, creating a potentially negative impact on nurse-to-patient ratios. In turn, sub-optimal staffing ratios may lead to less intensive monitoring and severe clinical deterioration of patients in the ICU or step-down unit [9]. Formal training and expertise in critical care, i.e. a “closed nursing model” is also a desirable feature for the nursing staff of an ICU/ the step-down unit.

Although we cannot make statistical inferences about the finding of mortality risk at the midpoint between reported outcomes in HICs and LMICs, we can enumerate likely health system and structural challenges to improving ICU outcomes. These factors, such as the “open model” discussed above, underscore important issues confronting most ICUs in LMICs.

Potentially important structural factors impacting outcomes among the critically ill in our institution include bed-allocation policies and capacity limitations. Thirty three percent of patients in the AKUHN ICU did not meet our institution’s ICU admission criteria and were admitted to ICU solely due to unavailability of beds in the general wards and in the step-down unit. Bed capacity limitations and allocation issues have a ripple effect in our institution and indeed in most ICUs in LMIC. Patients who do require ICU care are either not admitted to the ICU directly or receive a sub-optimal level of care in step-down units until a bed becomes available in the ICU. Predictably, such patients admitted from the step-down unit in our cohort had a particularly high mortality.

We can identify several limitations in our study. Firstly, this was a single center study and as such, the results may not be applicable across all patient populations in Kenya or other well-resourced ICUs in LMICs. This is largely because different hospitals have different ICU admission criteria, staffing ratios, management protocols and management policies. Secondly, we could not verify what proportion of critically ill patients stepped up to the ICU from the step-down unit were admitted to the step-down unit due to lack of an ICU bed, as data on the appropriateness of the initial step-down unit admission were unavailable. Thirdly, this was a retrospective study and it was difficult to gauge the diagnostic accuracy of the documented indications for admission from case notes and some case files were missing. However, the retrieval rate of complete case files at our institution was high (81.1%); higher than that in the public ICU study (67%) [5]. This limitation is unlikely to be addressed until reliably available and detailed paper or electronic records are in place. Additionally, the mortality rate in the cohort of critically ill patients for which we had only limited records was comparable to that in which a complete medical record was available (29.4% vs 32.4%), rendering our data robust, and our findings an accurate representation of outcomes in the AKUHN ICU.

## Conclusion

Our analysis of mortality prediction models concluded that 48-hour SOFA performed significantly better in predicting outcomes among critically ill patients admitted to the AKUHN ICU than admission SOFA, MPM-III and delta-SOFA. We found that mortality in this cohort was higher than we expected in this well-resourced ICU. Critically ill patients with advanced co-morbidities and individuals stepped-up from the step-down unit were unlikely to survive their ICU stay. Critical care capacity building efforts in other LMICs should be informed by the implications of this analysis. We suggest that achieving good outcomes in intensive care depends on more than the provision of world-class resources. Policies for fair allocation of beds, protocol-driven admission criteria and appropriate patient selection could contribute to lowering the risk of mortality among the critically ill to a level on par with well-resourced ICUs in HICs.

## Supporting information

S1 Data (XLSX)
